# Biointeractions of Herbicide Atrazine with Human Serum Albumin: UV-Vis, Fluorescence and Circular Dichroism Approaches

**DOI:** 10.3390/ijerph15010116

**Published:** 2018-01-11

**Authors:** Meiqing Zhu, Lijun Wang, Yu Wang, Jie Zhou, Jie Ding, Wei Li, Yue Xin, Shisuo Fan, Zhen Wang, Yi Wang

**Affiliations:** Key Laboratory of Agri-Food Safety of Anhui Province, School of Resources and Environment, Anhui Agricultural University, Hefei 230036, China; zhumeiqing@ahau.edu.cn (M.Z.); li_jun_wang@yeah.net (L.W.); wangyu@ahau.edu.cn (Y.W.); zhoujie314@ahau.edu.cn (J.Z.); djie@ahau.edu.cn (J.D.); liwei14@ahau.edu.cn (W.L.); xinyue@ahau.edu.cn (Y.X.); fanshisuo@ahau.edu.cn (S.F.); zwang@ahau.edu.cn (Z.W.)

**Keywords:** atrazine, fluorescence quenching, human serum albumin, spectroscopy

## Abstract

The herbicide atrazine is widely used across the globe, which is a great concern. To investigate its potential toxicity in the human body, human serum albumin (HSA) was selected as a model protein. The interaction between atrazine and HSA was investigated using steady-state fluorescence spectroscopy, synchronous fluorescence spectroscopy, UV-Vis spectroscopy, three-dimensional (3D) fluorescence spectroscopy and circular dichroism (CD) spectroscopy. The intrinsic fluorescence of HSA was quenched by the atrazine through a static quenching mechanism. Fluorescence spectra at two excitation wavelengths (280 and 295 nm) showed that the fluorescence quenched in HSA was mainly contributed to by tryptophan residues. In addition, the atrazine bound to HSA, which induced changes in the conformation and secondary structure of HSA and caused an energy transfer. Thermodynamic parameters revealed that this binding is spontaneous. Moreover, electrostatic interactions play a major role in the combination of atrazine and HSA. One atrazine molecule can only bind to one HSA molecule to form a complex, and the atrazine molecule is bound at site II (subdomain IIIA) of HSA. This study furthers the understanding of the potential effects posed by atrazine on humans at the molecular level.

## 1. Introduction

Atrazine (ATR) is a triazine herbicide with an excellent herbicidal activity. ATR can kill a broad spectrum of plants and has been widely used to control broadleaf and grass weeds in past decades [1,2,3]. ATR plays a key role in inhibiting the photosynthesis of plants by targeting the D1 protein of photosystem II (PSII) and causes the death of weeds to achieve the function of weed prevention and treatment [4,5,6,7,8]. Such herbicides have played a significant role in agriculture but can unfortunately enter surface waters through various paths and can further enter groundwater [9,10,11]. These compounds may remain in crops and can directly or indirectly enter the food chain and then the human body, potentially harming human health [12,13,14]. The use of ATR, simazine, prometryn, and terbutryn, as four classic types of triazine herbicides, has been limited by the European Parliament Agency. For example, ATR plays a significant role in inhibiting the specific binding of various types of proteins such as oestrogens and progesterone receptors; the extent of its harm varies and can even lead to cancer [15,16,17]. ATR may harm the central nervous systems, endocrine systems, immune systems, and reproductive systems of amphibians, rats, and pigs, and other animals, and ATR can produce genotoxic damage in fish [18,19,20]. However, up to now, there have been no studies regarding the interactions between ATR and physiologically important proteins using fluorescence spectroscopy; these studies are needed to provide important insight into the interaction of plasma proteins with ATR.

Human serum albumin (HSA) is one of the most abundant proteins in plasma, accounting for approximately 60% of the total serum protein content, and is an important carrier of transport proteins [21,22]. Approximately 80% of the blood osmotic pressure is provided by HSA. Many of the endogenous and exogenous substances that enter the human body bind to HSA through reversible noncovalent binding and are transported to various parts of the body after binding [23]. Therefore, HSA has important physiological functions, such as bodily protection and information storage, and is the main carrier through which various endogenous and exogenous substances are transported and eliminated [24]. The binding of small herbicide molecules with HSA will affect its structure and function, so it is important to study the interaction of herbicides with HSA to understand the metabolism and biological effects in vivo.

Many physical and chemical methods exist to determine the structure and function of bioactive molecules as well as their mechanism of action and other information. Some of these molecules emit optical signals and can therefore be determined spectroscopically [25,26]. Spectroscopy is often used to study the material interactions in a method and has many advantages, including its simple operation, ease of use, good detection performance, high sensitivity and wide selectivity. Spectroscopic techniques mainly include fluorescence spectroscopy, UV-Vis absorption spectroscopy, infrared spectroscopy, and circular dichroism (CD) spectroscopy, which are the commonly used methods for studying the interactions between small molecules and proteins [27,28]. 

In this study, we investigated ATR, and the interaction between small herbicidal molecule and proteins was mainly explored using fluorescence methods. The aim of this work was to study the potential influence of pesticide residues to the human body, and HSA was selected as the research object. The mechanism was elucidated at the molecular level to understand the effect on human health by studying the interaction of ATR and proteins.

## 2. Experimental Section

### 2.1. Materials

HSA without fatty acids (≥99.9%, A1887) and digitoxin (Dig, D5878) were purchased from Sigma-Aldrich (St. Louis, MO, USA). Flufenamic acid (FA, 186419) and phenylbutazone (PB, 451567) were obtained from J&K (Beijing, China) and were used without further purification. Three drugs, digitoxin, flufenamic acid and phenylbutazone, were used as labelled drugs for binding at sites I, II and III of HSA, respectively. The specific binding site of the ligand on the HSA can be determined by the labelling competition experiment. Metsulfuron-methyl, prometryn and ATR were purchased from ANPEL Laboratory Technologies Inc. (Shanghai, China), and stock solutions of these compounds were prepared using anhydrous ethanol. All other commercial reagents were obtained from J&K Scientific, Alfa Aesar (Ward Hill, MA, USA) and Sigma-Aldrich. A Tris-HCl buffer solution (pH 7.4) was prepared with 0.20 mol/L trihydroxymethylaminomethane and 0.10 mol/L hydrochloric acid. An HSA solution (3 × 10^−5^ M) was prepared in Tris-HCl buffer and was stored in the refrigerator at 4 °C. Other chemicals and reagents used in this study were all analytical grade. All water used in these experiments was deionized water. 

### 2.2. Instrumentation

The fluorescence spectra were measured on a 970 CRT fluorescence spectrophotometer (Shanghai Electronic Science Instrument Factory, Shanghai, China). An HH-6 digital constant temperature water bath (Ronghua Instrument Factory, Changzhou, China) was used for temperature control. The three-dimensional (3D) fluorescence spectra and the synchronous fluorescence spectra were measured on a Cary Eclipse fluorescence spectrophotometer (Agilent Technologies, Palo Alto, CA, USA). The UV spectra were recorded using a UV-1800 UV spectrophotometer (Shimadzu, Kyoto, Japan). All the above measurements were made using quartz cuvettes with a 1 cm path length and 3 mL volume. The pH of the buffer solution was measured using a PHS-25 digital pH metre (Shanghai REX Instrument Factory, Shanghai, China). The CD spectra were measured using a Jasco-810 spectrometer (Jasco, Tsukuba, Japan).

### 2.3. Steady-State Fluorescence Spectra

Steady-state fluorescence was determined using a 970CRT fluorescence spectrophotometer (Shanghai Electronic Science Instrument Factory, Shanghai, China) equipped with a 1.0 cm quartz cuvette. The temperature was controlled with an HH-6 digital constant temperature water bath. The excitation wavelength (λ_ex_) are set to 280 nm and 295 nm, respectively. And the maximum emission wavelength (λ_em_) of HSA were 340 nm and 350 nm, respectively. The emission spectrum was scanned in a wavelength range of 300–500 nm, and the excitation and emission slit widths were fixed at 5.0 nm. The scanning speed was 240 nm/min. The corresponding concentration of the Tris-HCl buffer was subtracted from all fluorescence spectra.

### 2.4. Fluorescence Titration Experiments

The 3 × 10^−5^ mol/L HSA stock solution was diluted to a 3 × 10^−6^ mol/L HSA solution. The ATR stock solution (6 × 10^−4^ mol/L), which was prepared in ethanol, was added continuously at three temperatures (298, 307 and 316 K), and the change in fluorescence intensity was measured by manual titration using a syringe (λ_ex_ = 280 nm and 295 nm, λ_em_ = 340 nm and 350 nm). The experimental temperature was controlled with a water bath that was had a digital constant temperature.

In solution, due to the inner filter effect, ATR may absorb some radiation generated by the fluorophore at the excitation or emission wavelength of HSA, or may influence excited radiation that reaches the fluorophore. To decrease the inner filter effects, the absorbance of the ligand at each concentration and the absorbance of the protein without ligand at the excitation and emission wavelength were recorded, respectively. The observed fluorescence intensity was corrected using the following equation:

(1) $$F_{\mathrm{cor}}=F_{\mathrm{obs}}e^{(A_{\mathrm{ex}}+A_{em})/2}$$

where *F*_cor_ and *F*_obs_ refer to the corrected and observed fluorescence intensities, respectively. *A_ex_* and *A_em_* represent the absorption of the ligand-protein solution at the excitation and the emission wavelengths, respectively. According to Makarska’s study [29], the intensity was obtained by deducting the ATR curve from the HSA-ATR spectra, at each step of titration with ATR.

### 2.5. Site Marker Competition Experiments

The binding sites of the ATR on the HSA were determined by adding three classical binding sites to create binding competition amongst competitive drugs (PB, FA and Dig) during the fluorescence titration. First, the three site markers were bound to HSA. Then, the ATR solution was gradually added to the mixture of the three markers and HSA, and the change in fluorescence intensity was measured.

### 2.6. UV-Vis Spectra

The 10^−6^ mol/L sample was diluted from a 10^−5^ mol/L HSA stock solution to which the ATR was added (from 0.3 × 10^−6^ mol/L to 2.4 × 10^−6^ mol/L at intervals of 0.3 × 10^−6^ mol/L). The mixed sample was placed in a 1.0 cm quartz cuvette, and the absorption spectra of the system at wavelengths of 200–500 nm were recorded using a UV-1800 UV spectrophotometer (Shimadzu, Kyoto, Japan) at room temperature (298 K) with a scanning speed of 300 nm/min. The background absorbance of the buffer solution was subtracted from the sample absorbance.

### 2.7. CD Spectra

The change in the secondary structure of HSA in the presence and absence of the ATR was measured using a Jasco-810 CD spectrometer under a constant nitrogen atmosphere in a quartz cuvette with a 0.1 cm path length over a scanning range of 200–260 nm. The step resolution was 0.1 nm, and the scanning speed was 50 nm/min. The value of the buffer solution was subtracted from all CD spectral data. The change in the secondary structure of HSA was calculated using the Jasco secondary structure estimation software that accompanied the spectrometer.

### 2.8. 3D Fluorescence Spectra

3D fluorescence spectra were scanned on a Cary Eclipse fluorescence spectrophotometer (Agilent Technologies, Palo Alto, CA, USA) using a quartz cuvette with a 1-cm path length and a 3-mL volume. The initial excitation wavelength was set to 200 nm, the emission wavelength scanning range was 200–500 nm, the *E*_x_ and *E*_m_ slit widths were both 10 nm, and the scanning frequency was 16.

### 2.9. Synchronous Fluorescence Spectra

Synchronous fluorescence spectra were scanned on a Cary Eclipse fluorescence spectrophotometer (Agilent Technologies) using a quartz cuvette with a 1-cm path length and a 3-mL volume. The HSA concentration was 3 × 10^−6^ mol/L, and the ATR solution was added to the HSA solution at concentrations ranging from 0 to 2.4 × 10^−6^ mol/L; λ_ex_ = λ_em_. When Δλ = 15 nm, the scan range was set to 260–320 nm, and when Δλ = 60 nm, the scanning range was 240–320 nm.

## 3. Results and Discussion

### 3.1. Study of the Quenching Mechanism of HSA by ATR

**Figure 1:**
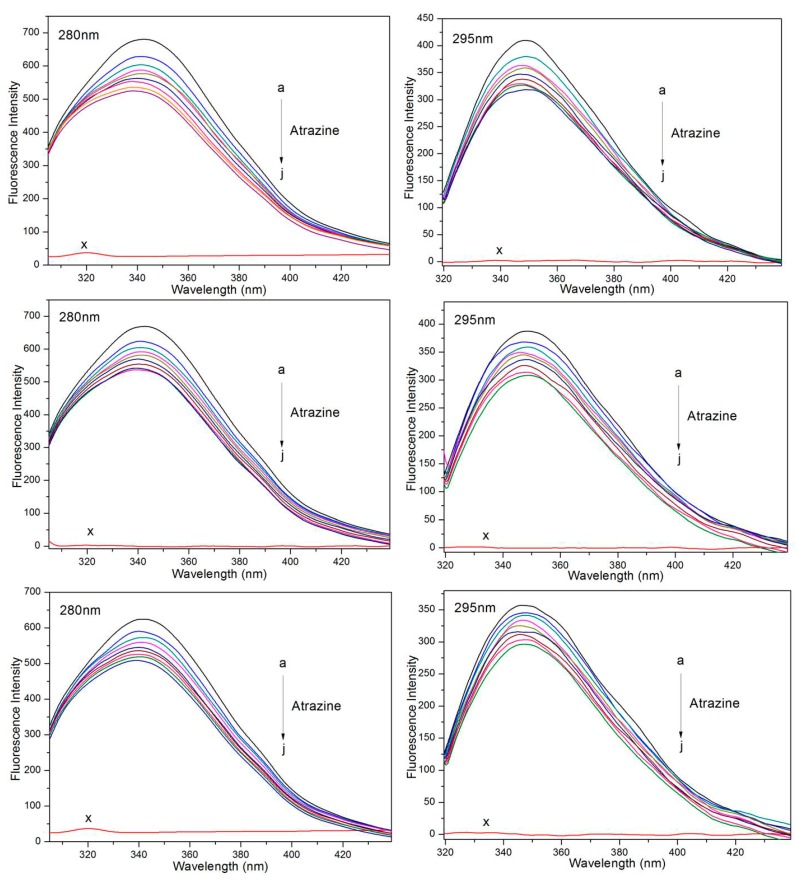
Steady-state fluorescence spectra of HSA in the presence of different concentrations of ATR at three different temperature (298 K, 307 K and 316 K). (a–j) 0, 0.3, 0.6, 0.9, 1.2, 1.5, 1.8, 2.1, and 2.4 × 10^−6^ mol/L respectively. (x) only ATR.

HSA produces UV fluorescence because it contains three amino acids with aromatic ring structures, including tryptophan (Trp), tyrosine (Tyr) and phenylalanine (Phe) [30,31]. Among them, Phe fluorescence is not often observed, so Trp and Tyr are commonly used as endogenous HSA fluorescent probes. When λ_ex_ = 280 nm, the fluorescence is mainly contributed to by Trp and Tyr residues, and when the excitation wavelength is 295 nm, only the fluorescence of the Trp residue is excited [32,33,34]. In this experiment, 280 nm and 295 nm were used as excitation wavelengths. Under the experimental conditions, the fluorescence emission spectrum of the HSA solution at a certain concentration was scanned. Under the same conditions, the fluorescence spectra of mixtures of HSA with different concentrations of ATR were scanned. The results are shown in Figure 1. For the ATR-HSA system, with an increase in the ATR concentration, the fluorescence intensity of the HSA emission peak decreased, indicating an interaction between the ATR and HSA and quenching of the endogenous fluorescence of HSA. Compared to the results of excitation at 280 nm and excitation at 295 nm, the fluorescence intensity of the Trp groups is more pronounced than the Tyr groups that are present in HSA, indicating that Trp is mainly involved in the quenching of HSA.

The mechanism by which small molecule ligands quench the fluorescence of plasma albumin is mainly divided into dynamic quenching and static quenching. In dynamic quenching, the excited molecules of the fluorescent substance collide with the quencher molecules, and through energy transfer or charge transfer, the excited molecules lose their excitation energy and return to the ground state without emitting photons. In static quenching, the quenching agent and fluorescent substance in the ground state form a complex, leading to fluorescence quenching. The static and dynamic quenching mechanisms follow the Stern–Volmer equation [35,36,37]:

(2) $$F_{0}/F=1+K_{SV}[Q]=1+K_{q}\tau_{0}[Q]$$

where *F*_0_ and *F* are the fluorescence intensity of the system without and with the quencher, respectively; *K_q_* is the quenching rate constant of the biomolecule; τ_0_ is the average life of the biomolecule in the absence of the quencher (for most biomolecules, τ_0_ is approximately 10^−8^ s^−1^) [38]; [*Q*] is the concentration of the quencher; and *K_SV_* is the quenching constant as the ratio of the bimolecular quenching rate constant to the single molecule decay rate constant. The Stern–Volmer diagram of the ATR-HSA system at 298 K can be calculated from the Stern–Volmer equation and the Stern–Volmer quenching constant for the interaction between ATR and HSA at three different temperatures (Table 1).

**Figure 2:**
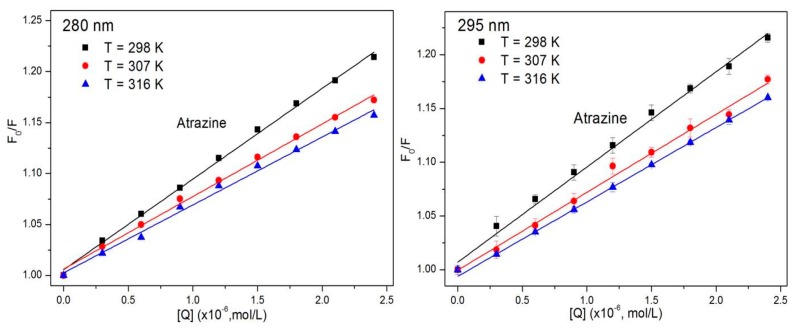
Stern–Volmer plot describing HSA fluorescence quenching caused by ATR at different excitation wavelengths. Data are mean ± SE (bars) (*n* = 3) (T = 298 K, 307 K, 316 K).

As shown in Figure 2, the Stern–Volmer diagrams of the ATR-HSA system have very high linear correlation coefficients, and it is speculated that the ATR-HSA combination has only one quenching mechanism. Table 1 lists the value of *K_q_* for different excitation wavelengths. The *K_q_* ranges from 6.66 × 10^12^ to 8.90 × 10^12^ mol/L at an excitation wavelength of 280 nm, and when the excitation wavelength set to 295 nm, the value of *K_q_* ranges from 6.07 × 10^12^ to 8.12 × 10^12^ mol/L. It is apparent from the data that the quenching constants at different excitation wavelengths are very close, indicating that ATR mainly quenches the fluorescence of Trp residues and is more than 100 times the maximum dispersion collision quenching constant (2.0 × 10^10^ mol/L) [39,40] of the various quenchers and biological macromolecules. Therefore, this reaction produces a complex of ATR and HSA, and the endogenous fluorescence of HSA is quenched via the static quenching mechanism. 

### 3.2. UV-Vis Spectra of ATR Interacting with HSA

**Figure 3:**
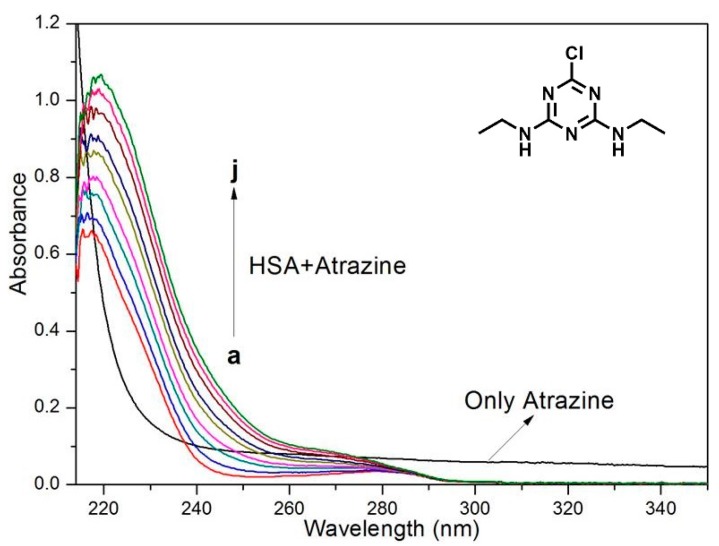
Absorption spectra of HSA (3.0 × 10^−6^ mol/L) upon the addition of ATR. The arrows show the increase in the ATR concentration. The ATR concentrations were 0.3, 0.6, 0.9, 1.2, 1.5, 1.8, 2.1 and 2.4 × 10^−6^ mol/L, respectively.

UV-Vis absorption spectroscopy is used to explore the structural changes and morphologies of complexes [41,42,43]. Dynamic quenching only affects the excited state of the fluorophore; therefore, dynamic quenching should not change the absorption spectrum. For static quenching, the changes in the UV-Vis absorption spectrum are due to the formation of the complex [44,45,46,47,48]. Figure 3 shows the absorption spectra of HSA after the addition of the ATR. The absorbance of HSA increased with the addition of different amounts of herbicides, accompanied by a 5-nm redshift. These data confirm that the spectral change is due to the formation of the complex and verifies the previous conclusion that the quenching mechanism of HSA and ATR is static quenching.

### 3.3. Study of the Binding Sites and Binding Number

**Figure 4:**
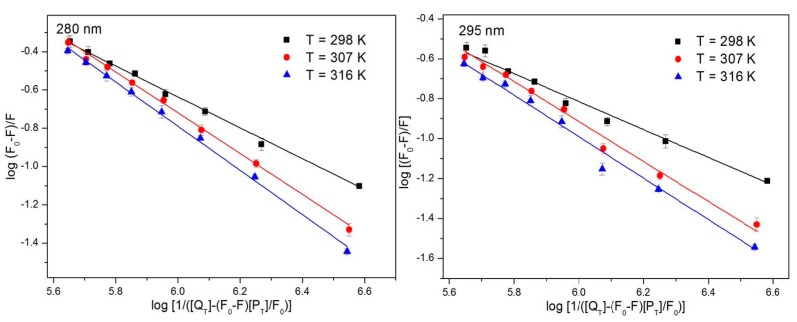
The log(*F*_0_*−F*)/*F* against log[1/([*Q_T_*]−(*F*_0_−*F*)[*P_T_*]/*F*_0_)] plots of ATR-HSA system at 298 K, 307 K and 316 K (*n* = 3).

For static quenching, data from the fluorescence spectra can be utilized to calculate the binding constant (*K_a_*) and number of binding sites (*n*) using the following formula [49]:

(3) $$\log\left[\left(F_{0}-F\right)/F\right]=n\log K_{a}+n\log[1/([Q_{T}]-\left(F_{0}-F)[P_{T}]/F_{0}\right)]$$

where *F* and *F*_0_ represent the fluorescence intensity with and without the quencher, respectively; *n* is the number of binding sites; *P_T_* is the total protein concentration; *Q_T_* is the total quencher concentration; and *K_a_* is a binding constant. *n*log*K_a_* is the intercept of the linear equation represented by $\log\left[\left(F_{0}-F\right)/F\right]$ and $\log[1/([Q_{T}]-\left(F_{0}-F)[P_{T}]/F_{0}\right)]$. Figure 4 is a plot of log [(*F*_0_−*F*)*/F*] versus log *K_a_*, and *n* can be calculated based on Equation (2). *K_a_* decreases with increasing temperature; therefore, the decrease in the stability of the complex that formed from the ATR and HSA at a relatively high temperature is not conducive to the binding, which is also a characteristic of static quenching. *K_a_* is large, which indicates a strong interaction of ATR with HSA. As shown in Table 2, the results at different excitation wavelengths are similar and the value of *n* is close to 1, which indicates that only one ATR molecule combines with one HSA molecule. This reaction proceeds at a ratio of 1:1.

Small molecule and protein binding sites can be confirmed by competitive marker experiments, providing a fast, simple and effective method [50,51,52]. To determine the binding sites of ATR on HSA, PB, FA and Dig were added to the ATR-HSA system as labelled drugs for binding at sites I, II and III of HSA, respectively. The binding constants of ATR with HSA at 298 K that were calculated using Equation (2) are listed in Table 3. It is apparent from the data in Table 3 that after the addition of PB and Dig to the ATR-HSA system, the binding constant *K_a_* did not change much. However, the binding constant of the ATR-HSA system greatly changed after the addition of FA. From the above conclusions, we know that the binding ratio of ATR to HSA is 1:1; therefore, we can speculate that the binding site of ATR on HSA is the same as that of fluoride on HSA. These results suggest that the binding site of ATR is located within site II (subdomain IIIA).

### 3.4. Combination Distance between ATR and HSA

**Figure 5:**
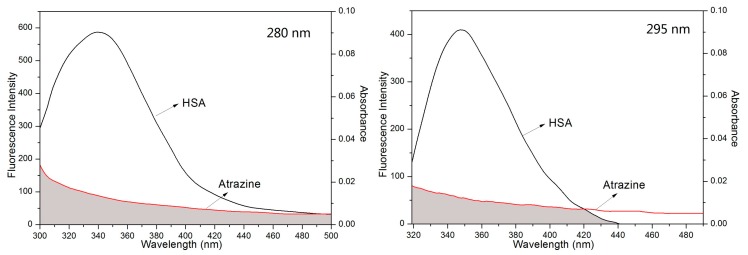
Overlap of the fluorescence spectrum of HSA and the absorption spectra of the ATR. C (HSA) = c (ATR) = 3.00 × 10^−6^ mol/L; T = 298 K.

The fluorescence intensity of HSA also decreased with the addition of ATR, indicating an energy transfer between the amino acid residues in HSA and ATR. The binding distance of the ATR-HSA system can be calculated by separately overlapping the UV-Vis spectra of ATR with the HSA fluorescence spectrum. The overlapping plots at different excitation wavelengths are shown in Figure 5.

According to Förster’s theory of nonradiative energy transfer [53], the energy transfer rate depends on three factors including the relative orientation of the donor and acceptor dipole, the overlap of the fluorescence emission spectrum of the donor with the absorption spectrum, and the distance between the donor and acceptor. The energy transfer effect is related not only to the distance between the receptor and the donor but also to the critical energy transfer distance *R*_0_, as described in the following equation:

(4) $$E=1-\frac{F}{F_{0}}=\frac{R_{0}{}^{6}}{R_{0}{}^{6}+r^{2}}$$

where *F* and *F*_0_ are the fluorescence intensities of HSA in the presence and absence of the drug, respectively, *r* is the binding distance between HSA and ATR, and *R*_0_ is the critical distance when the energy transfer is 50%. *R*_0_ can be calculated using the following formula:

(5) $$R_{0}{}^{6}=8.8\times 10^{-25}K^{2}N^{-4}\phi J$$

where *K*^2^ is the spatial orientation factor between the emission dipole of HSA and the absorption dipole of the ATR. In general, the value of *K*^2^ is 2/3 *N* and is the refractive index of the medium, with a value of 1.336. *ϕ* is the quantum yield of HSA in the presence of ATR, and *J* is the overlap integral of the fluorescence emission spectra of HSA with the absorption spectra of the ATR. *J* can be calculated from the following formula:

(6) $$J=\frac{\sum F(\lambda )(\lambda )\lambda 4\Delta \lambda }{\sum F(\lambda )\Delta \lambda }$$

where *F*(*λ*) is the fluorescence intensity of the HSA solution at the specified wavelength, and *ε*(*λ*) is the molar absorption coefficient of the solution of ATR. *J* can be evaluated from the spectrum in the plot. The *E*, *J*, *R* and *r* values of the ATR-HSA system at different excitation wavelengths are listed in Table 4. The value of *r* in the presence of the drugs was less than 8.0 nm at different excitation wavelengths, which indicates that the quenching of HSA by ATR is consistent with the nonradiative energy transfer theory and potentially indicates that the combination of ATR effectively quenches the endogenous fluorescence of HSA through energy transfer. Through the overlapping points of results from different excitation wavelengths, it is apparent that the largest contribution of endogenous fluorescence of HSA is Trp residues.

### 3.5. Thermodynamic Parameters and Binding Modes

**Figure 6:**
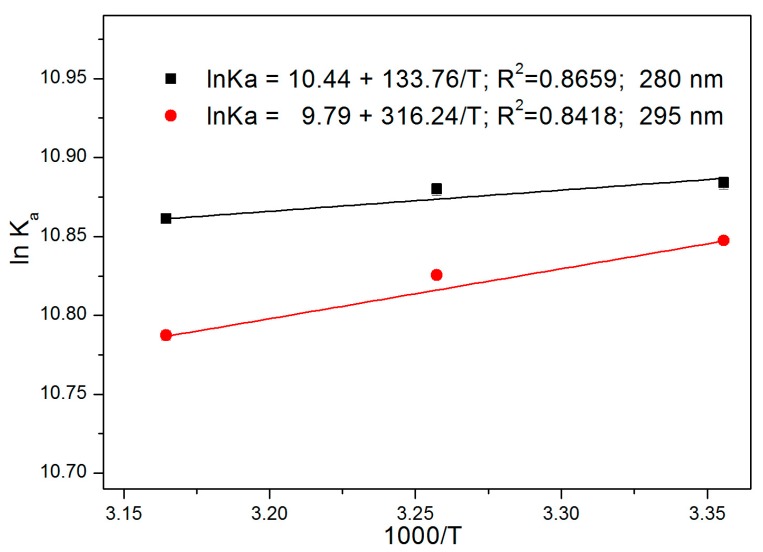
Van’t Hoff plots for the ATR-HSA systems.

The interactions between small molecules and proteins are weak and include four different forces and modes of action: hydrogen bonds, electrostatic interactions, hydrophobic effects and van der Waals forces. Based on a large number of experimental results, Ross [54,55] and others summed the parameters of the interactions of proteins with small molecules, such as the binding force properties and the thermodynamics of the interaction. Their results indicated that hydrophobic forces play a major role when Δ*S* > 0 and Δ*H* > 0, and hydrogen bonds and van der Waals forces play a dominant role in the binding process when Δ*S* < 0 and Δ*H* < 0. The condition of Δ*H* < 0 and Δ*S* > 0 corresponds to electrostatic interactions. Using the van’t Hoff equation, the thermodynamics of the interactions between small molecules and proteins can be determined according to the change in the binding constant at different temperatures, and the type of interactions between small molecules and proteins can be assessed:

(7) $$\ln K_{a}=-\Delta H/RT+\Delta S/R$$

where *K_a_* is the binding constant calculated from Equation (2), *T* is the absolute temperature, and *R* is the gas constant. According to the van’t Hoff equation, the values of Δ*H* and Δ*S* can be obtained from the graphs of 1/*T* versus ln *K_a_* (Figure 6), where Δ*H* and Δ*S* are the slope and intercept in the van’t Hoff formula, respectively. The free energy change (Δ*G*) is then determined according to the following equation:

(8) ΔG=ΔH−TΔS

where Δ*G* is negative, indicating that the binding of ATR to HSA in solution is spontaneous (Table 2). The Δ*S* of the three ligands is positive, which indicates that the main interaction pattern between ATR and HSA is hydrophobic, whereas Δ*H* is negative, indicating that hydrogen plays a key role in the interaction between ATR and HSA. Based on the above results, hydrophobic forces and hydrogen bonding are the main modes of interaction between ATR and HSA.

### 3.6. Study of the Synchronous Fluorescence Spectra

Synchronous fluorescence spectroscopy has many advantages and is often used to characterize complex mixtures; this technique can also be used to investigate changes in protein conformation [56]. The intrinsic fluorescence of HSA is mainly derived from Tyr and Trp residues [57]. In this experiment, synchronous fluorescence spectroscopy was used to assess the effect of metsulfuron-methyl, prometryn and ATR on the molecular conformation of HSA. When ∆*λ* was set to 15 nm, the synchronous fluorescence spectrum contained features characteristic of Tyr residues in HSA. When the distance between the excitation and emission wavelengths was set to 60 nm, the resulting synchronous fluorescence spectrum contained characteristics of Trp residues in HSA.

**Figure 7:**
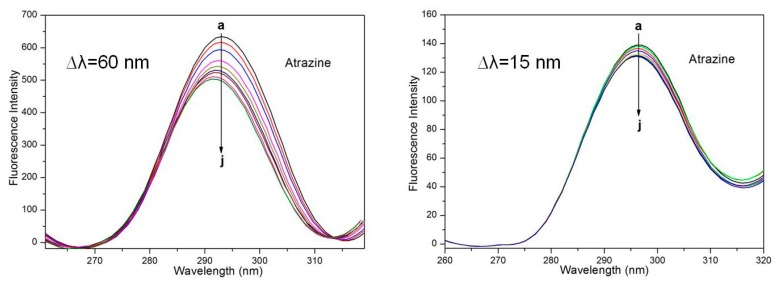
Synchronous fluorescence spectra of HSA with various amounts of ATR.

Figure 7 shows the change in the HSA synchronic fluorescence spectrum after the addition of ATR. When ∆*λ* = 60 nm, the synchronous fluorescence intensity decreased with increasing ATR concentration, and the maximum emission wavelength was a 4-nm blueshift; these results indicate that the histidine residues underwent conformational changes, polarity decreased, and hydrophobicity increased. When ∆*λ* = 15 nm, although the fluorescence intensity decreased with the increase in the ATR concentration, there was no obvious blueshift or redshift, and the quenching at ∆*λ* = 60 nm was better than at ∆*λ* = 15 nm. The above results indicate that ATR has no effect on the microenvironment of the Tyr residues but has an effect on the microenvironment of the Trp residues in HSA. In addition, these data indicate that ATR is bound closer to the Trp residue than to the Tyr residue and that the binding site is predominantly distributed over the Trp moiety.

### 3.7. Conformational Changes of HSA Detected by CD and 3D Fluorescence Spectroscopy

**Figure 8:**
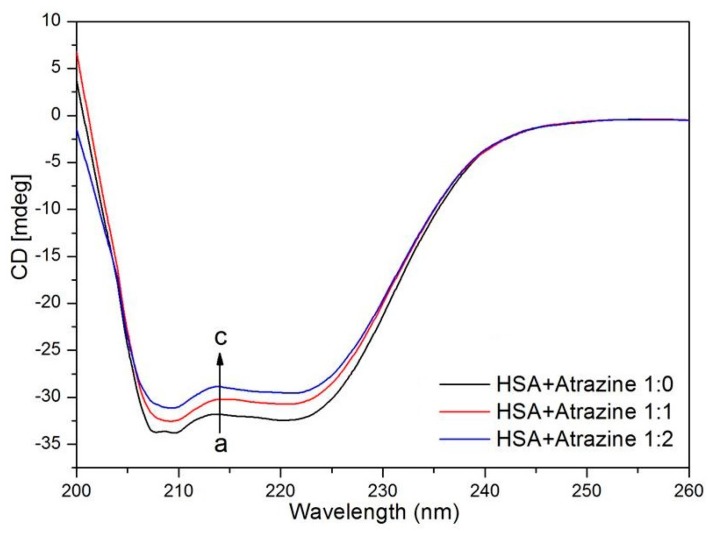
Far-UV CD spectra of HSA in the presence of different concentrations of ATR.

CD spectroscopy is widely used to study the interactions between small molecules and proteins because of its high sensitivity and because it is a very effective method to analyse the secondary structure of proteins [58,59,60]. Figure 8 shows CD spectra over an interval of 200–260 nm with and without ATR. The CD spectra of HSA have negative peaks at 208 nm and 220 nm, which are characteristic of an α-helix in the protein. With the continuous addition of ATR, the values of the CD spectra increased; that is, after the addition of ATR, the HSA structure changed. Table 5 lists the percentages of α-helixes, β-sheets, β-turns and random coils that were calculated using the CDSSTR algorithm in the CDPro package. α-helixes decreased as the ATR concentration increased, which indicates that the ATR has a direct effect on the amino acid residues of HSA, which leads to the destabilization of the spatial protein structure and to spiral structure extension. Agreeing with the FT-IR spectroscopy results of the Purcell’s study, it was shown that the binding of ATR to HSA results in a change in the conformation of HSA [61].

**Figure 9:**
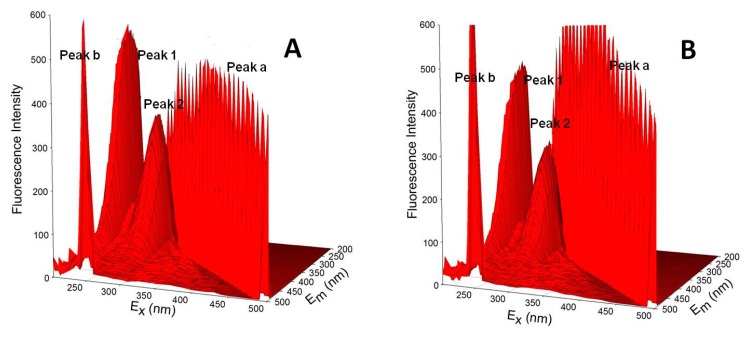
Three-dimensional fluorescence spectra of free HSA (**A**) and HSA complexed with the ATR (**B**).

Three-dimensional fluorescence spectroscopy is an effective method to comprehensively investigate protein structure [62]. The 3D fluorescence spectra are used to investigate the interference of ATR with the HSA spatial structure, which can reflect the structural changes of HSA. Table 6 lists 3D fluorescence characteristics. Figure 9 shows 3D fluorescence spectra of the ATR-HSA system. (λ_ex_ = λ_em_) is the Rayleigh scattering peak, peak b (λ_em_ = 2λ_ex_) is the secondary scattering peak, and peak 1 mainly expresses the spectral characteristics of the Tyr and Trp residues. Because the excitation wavelength was λ_ex_ = 280 nm, the main excitation was related to the fluorescence of the Tyr and Trp residues, and the Phe residue fluorescence was negligible. Peak 2 mainly reflects the fluorescence signal of the polypeptide chain skeleton structure of the protein (for example, C = O). When the ATR are present, the fluorescence intensity of peak 2 decreases, indicating that the HSA peptide chain structure changed, which is consistent with the CD results. The fluorescence intensities of peaks 1 and 2 decreased, verifying the results of synchrotron fluorescence and CD. These results show that the addition of ATR exposed some hydrophobic residues to the water environment, resulting in a conformational transformation of the protein, i.e., a transition from a compact structure to a loose state in which the active portion of the protein was lost.

## 4. Conclusions

The interactions between ATR and HSA were studied using steady-state fluorescence spectroscopy at different excitation wavelengths, and the fluorescence quenching mechanism was determined for static quenching. We verified that the fluorescence quenched during the binding of ATR to HSA was mainly contributed to by Trp residues in HSA. The changes in the HSA conformation and secondary structure were verified using UV-Vis, 3D fluorescence, synchronous fluorescence, and CD spectroscopy. Based on the above data, ATR changes the conformation and secondary structure of HSA by combining at site II on HSA, and ATR and HSA (Trp) undergo energy transfer during binding, based on the calculations of the bond distance. The thermodynamic parameters indicated spontaneous combinations of ATR and HSA. ATR and HSA form a complex in a ratio of 1:1, primarily through electrostatic interactions. As ATR and HSA form a complex, fluorescence quenching occurs. The results of this study provide useful information for the use of ATR while minimizing its potential effects on humans.

## Figures and Tables

**Table 1:** Stern-Volmer quenching constants for the interactions of ATR with HSA at different temperatures.

Compound	T (K)	*K_SV_* (×10^4^·M^−1^)	*Kq* (×10^12^·M^−1^·s^−1^)	*R* ^a^	*SD* ^b^
ATR (280 nm)	298	8.90	8.90	0.9975	0.0226
	307	7.12	7.12	0.9957	0.0235
	316	6.66	6.66	0.9934	0.0274
ATR (295 nm)	298	8.12	8.12	0.9990	0.0147
	307	6.83	6.83	0.9984	0.0184
	316	6.07	6.07	0.9989	0.0239

**^a^** *R* is the correlation coefficient. **^b^** *SD* is the standard deviation of the *K_SV_* values.

**Table 2:** Binding constants and thermodynamic parameters of the ATR to HSA.

Compound	T (K)	*K_a_* (×10^4^ L mol^−1^)	*n*	*R* ^a^	Δ*H* (kJ·mol^−1^)	Δ*G* (kJ·mol^−1^)	Δ*S* (J·mol^−1^·K^−1^)
ATR (λ_ex_ = 280 nm)	298	5.33 ± 0.01	1.0434	0.9986		−26.97	
	307	5.31 ± 0.03	0.9647	0.9961	−1.11	−27.75	86.79
	316	5.21 ± 0.02	0.8743	0.9949		−28.54	
ATR (λ_ex_ = 295 nm)	298	5.14 ± 0.02	0.9874	0.9978		−26.88	
	307	5.13 ± 0.02	0.9437	0.9965	−2.63	−27.62	81.39
	316	4.84 ± 0.03	0.8256	0.9984		−28.35	

**^a^** *R* is the correlation coefficient.

**Table 3:** Effects of the site probe on the binding constants of ATR to HSA.

Compound	Site Marker	*K* (×10^4^ L mol^−1^)	*R* ^a^
ATR (λ_ex_ = 280 nm)	Blank	5.33 ± 0.04	0.9986
	PB	5.41 ± 0.02	0.9948
	FA	2.09 ± 0.01	0.9972
	Dig	5.19 ± 0.02	0.9831
ATR (λ_ex_ = 295 nm)	Blank	5.14 ± 0.02	0.9978
	PB	4.98 ± 0.03	0.9932
	FA	1.93 ± 0.02	0.9918
	Dig	5.17 ± 0.03	0.9938

**^a^** *R* is the correlation coefficient.

**Table 4:** Parameters of *E*, *J*, *R*_0_ and *r* of ATR-HSA system.

Compound	*E* (%)	*J* (cm^3^ L mol^−1^)	*R*_0_ (nm)	*r* (nm)
ATR (λ_ex_ = 280 nm)	5.8739	1.6383 × 10^−14^	2.7638	4.3871
ATR (λ_ex_ = 295 nm)	4.2154	1.0895 × 10^−14^	1.9324	3.3289

**Table 5:** Conformational changes in the secondary structure of HSA in the presence and absence of ATR.

Sample	Secondary Structure (%)			
	α-Helix	β-Sheet	β-Turn	Random Coil
HSA	32.5	8.3	28.2	31.0
HSA + ATR (1:1)	30.3	8.5	26.3	34.8
HSA + ATR (1:2)	27.3	5.2	28.8	38.6

**Table 6:** Intensity ratios of peaks 1 and 2.

Compound	Peak	Peak Position λ_ex_/λ_em_ (nm/nm)	Stokes Δ*λ* (nm)	Intensity F
HSA	Peak 1	230/320	90	594
	Peak 2	280/330	50	387
HSA-ATR	Peak 1	230/320	90	519
	Peak 2	280/330	50	321
